# Pseudoarthrosis repair after failed metatarsophalangeal 1 arthrodesis: Turnaround inlay graft in 26 patients

**DOI:** 10.3109/17453674.2011.552782

**Published:** 2011-02-10

**Authors:** Imre M Takács, Bart A Swierstra

**Affiliations:** Department of Orthopaedic Surgery, Sint Maartenskliniek, Nijmegen, The Netherlands

We present an inlay bone grafting technique for the treatment of failed first metatarsophalangeal joint (MTP-1) arthrodesis. To our knowledge, this technique has not been described before for this specific condition.

## Technique

**Figure 1.**
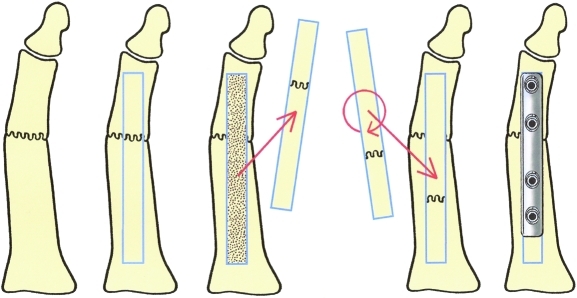
Schematic representation of turnaround inlay graft for MTP 1 pseudarthrosis.

The patient is placed in supine position with a tourniquet around the upper or lower leg. An incision is made over the dorsum of the first ray. The pseudoarthrosis is exposed. Existing hardware is removed when necessary. A corticocancellous 0.5–0.75 × 5 cm bone block, including healthy metatarsal bone and the pseudoarthrosis site, is osteotomized and mobilized. The bone block is turned around 180 degrees and put back into its bone bed, bridging the pseudoarthrosis site with a continuous piece of healthy bone. Fixation is done with a plate (AO third tube) and screws (Figure 1). The patient is mobilized with heel weight bearing for 8 weeks.

## Patients

Between 2001 and 2009, 26 patients (26 feet) with pseudoarthrosis of a MTP-1 arthrodesis were operated with this technique. Their mean age at operation was 63 (52–76) years. The previous fixation method was most often with screws, or with a K-wire or a plate. The average time from the last arthrodesis attempt until revision arthrodesis was 12 (4–31) months. The average time to follow-up was 4.5 (0.5–9) years. At follow-up, all patients were sent a Foot Function Index (FFI) form and a questionnaire containing a VAS score for pain, a question about satisfaction, and a question about orthopedic footwear.

**Figure 2.**
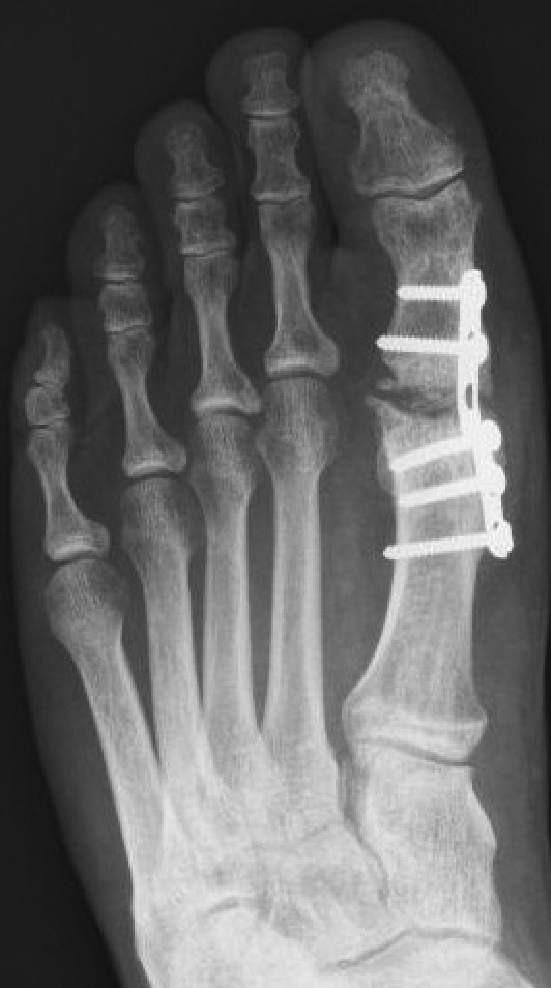
MTP-1 pseudarthrosis after removal of hemiprosthesis.

**Figure 3.**
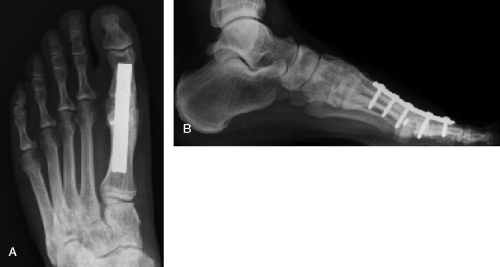
A and B. 6 months after turn around inlay graft.

25 of 26 patients returned the questionnaire. The average postoperative FFI score was 23 (0–47). The average postoperative VAS score for pain was 2.5 (0–9). 21 patients were satisfied with the postoperative result. 10 patients used prescription shoes. 2 patients presented with plate breakage because of non-union at 10 months and at 5 years after surgery, but they did not have pain and did not need treatment. In the other 24 patients, there was no sign of non-union at discharge from routine follow-up (Figures 2 and 3). In 2 patients, hardware was removed because of irritation. 1 patient had a relapse of Sudeck dystrophy. 1 patient had a correction osteotomy because of malposition of the hallux 6 years after surgery.

## Discussion

Pseudoarthrosis after attempted MTP-1 arthrodesis occurs in 1–14% of cases (Coughlin and Shurnas 2003, Coughlin et al. 2005, Gaucher and Coughlin 2006, Bennet and Sabatta 2009). Hope et al. (2010) described acceptable outcome scores with only removal of material and debridement in 7 of 11 patients. In our series, the 2 patients with hardware failure did not want further treatment, which confirms that some patients tolerate a non-union well. Hamilton et al. (2009) described other surgical treatment options. A Keller resection arthroplasty has several mechanical disadvantages and is not recommended for active patients. Resection of the pseudoarthrosis and refixation will lead to shortening of the first ray. This might be overcome with a bone block distraction arthrodesis. Fusion after this procedure varied from 79–92% in small series of patients with different indications (Hecht et al. 1997, Brodsky et al. 2000, Meyerson et al. 2000). Our fusion rate in 23/25 patients is similar or better, but our technique is less demanding.

Our technique has some advantages. Because no additional incision for graft harvesting is needed, there is no donor site morbidity. As the surgical approach is only from the dorsal side, disruption of local vascularization is limited, which is beneficial for fusion. The alignment of the hallux remains unchanged. But if the position is unsatisfactory, small corrections are possible without complete disruption of the pseudarthrosis. A disadvantage of the technique is the sometimes difficult radiographic judgment of the fusion of the graft. The patients with the plate breakage were already discharged from routine postoperative follow-up and were satisfied for 10 months and 5 years, respectively, but apparently the graft was not incorporated. Despite satisfaction in 21/25 patients, the mean FFI of 23 (0–49) is not as good as our results after primary MTP-1 fusion with an FFI of 8 (0–59) (Van Doeselaar et al. 2010).
